# Sleep is the best medicine: assessing sleep, disordered eating, and weight-related functioning

**DOI:** 10.1007/s40519-023-01610-y

**Published:** 2023-11-22

**Authors:** Rachel D. Barnes, Brooke Palmer, Sheila K. Hanson, Jessica L. Lawson

**Affiliations:** 1 Division of Geriatrics, Palliative and Primary Care, University of Minnesota Medical School, MMC 741, 420 Delaware Street SE, Minneapolis, MN 55455 USA; 2 Department of Psychology, School of Entrepreneurship and Management, University of North Dakota, Grand Forks, ND USA; 3 VA Connecticut Healthcare System, West Haven, CT USA; 4 Department of Psychiatry, Yale School of Medicine, New Haven, CT USA

**Keywords:** Loss of control eating, Overweight, Community sample, Sleep duration, Depression, Body mass index

## Abstract

Sleep quality is linked to disordered eating, obesity, depression, and weight-related functioning. Most research, however, has focused on clinical populations. The current study investigated relationships between sleep quality, disordered eating, and patterns of functioning in a community sample to better understand relationships among modifiable health behaviors. Participants (*N* = 648) recruited from Amazon Mechanical Turk completed assessments of eating, depression, weight-related functioning, and sleep. Self-reported height and weight were used to calculate body mass index (*M* = 27.3, *SD* = 6.9). Participants were on average 37.6 years (*SD* = 12.3), primarily female (65.4%), and White, not Hispanic (72.7%). Over half of participants endorsed poor sleep quality, and average sleep scores were above the clinical cutoff for poor sleep quality. Sleep scores were significantly positively correlated with disordered eating, depression, and weight-related functioning, even after adjusting for age, body mass index, and sex. Multivariate regression models predicting weight-related functioning and depression showed that both sleep quality and disordered eating independently predicted depression. Sleep quality did not independently predict weight-related functioning; however, disordered eating did. To the best of our knowledge, this is the first study to assess sleep behaviors, disordered eating, and weight-related functioning in a community sample of weight diverse participants. Results indicate that most participants endorsed poor sleep quality, which was associated with disordered eating patterns, including binge eating and poorer weight-related functioning, even after controlling for body mass index, highlighting that this relationship exists across the weight spectrum. These results speak to the importance of health behavior assessment and intervention within nonclinical samples.

*Level of evidence* Level III: evidence obtained from well-designed cohort or case–control analytic studies

## Introduction

A bidirectional relationship exists between aspects of sleep, such as duration, quality, efficiency, timing, and variability, and weight gain and obesity [1]. Pathways between poor sleep and overweight or obesity may exist due to neurochemical changes impacting hunger, autonomic nervous system dysregulation, and/or having more time to eat if awake [2, 3]. Similarly, comorbidities of obesity impact sleep quality. These factors include diagnoses, such as obstructive sleep apnea, gastroesophageal reflux disease, and degenerative joint disease [1]. Poor sleep quality and excess weight, as defined by body weight associated with a body mass index (BMI; kg/m^2^) in the overweight or obese range, also are related to important variables, such as worse quality of life [4, 5], depression [6–8], and the way in which weight impacts domains of functioning.

Excess weight also is linked to disordered eating patterns, including loss of control (LOC) eating and binge eating [9–11]. Experiencing a subjective loss of control while eating is part of the diagnostic criteria for an objective binge eating episode (OBE) according to the *Diagnostic and Statistical Manual of Mental Disorders-fifth edition* (*DSM-5)* [12]. At least weekly OBEs are required for a *DSM-5* diagnosis of binge eating disorder (BED) or bulimia nervosa (BN) [12]. Individuals also may experience loss of control eating in the absence of objectively large amounts of food, referred to as subjective binge eating episodes (SBE). While not factored into an eating disorder diagnosis of BED or BN, SBEs are clinically relevant due to their association with disordered eating pathology and emotional- and eating-related distress [13, 14]. Loss of control eating, regardless of food amount consumed, also is associated with poorer quality of life in college women [15], community samples [16], and clinical samples, such as individuals with disordered eating pursuing weight-loss surgery [17].

Despite the relationships between excess weight and sleep and disordered eating [1, 9], potential associations between disordered eating and sleep remain under-investigated with inconsistent findings. Existing research typically has been conducted among individuals with anorexia nervosa [18] or in other treatment-seeking clinical populations with eating disorders [19, 20] as highlighted by comprehensive reviews [21, 22], a meta-analysis [18], or following weight-loss surgery [23]. The literature suggests relationships between different forms of disordered eating diagnoses (e.g., anorexia nervosa) and poor sleep [18]. Da Luz and authors conducted a systematic review [24] of studies including clinical and community participants with binge eating behaviors, finding that participants with binge eating behaviors exhibited poorer sleep quality compared to participants with no binge eating patterns; however, the authors did caution lack of validated sleep measures as a limitation within the literature. In a clinical sample of individuals with loss of control eating following weight-loss surgery, participants completed measures of sleep quality, disordered eating, health-related quality of life, and physical and mental health functioning [23]. Lawson et al. [23] results showed participants with poor sleep quality reported higher levels of disordered eating compared to those who reported good sleep quality. Regression analyses revealed that disordered eating predicted physical but not mental health functioning and sleep predicted mental but not physical health functioning. While problematic sleep [25] and disordered eating behaviors [26] also are quite common within nonclinical samples, the possible relationship between disordered eating and sleep has been largely overlooked in the general population.

Existing nascent research suggests potential associations between disordered eating and sleep even within the broader population. Yeh & Brown [27] investigated the relationship between sleep quality and BMI in a community sample and found that binge eating severity and night eating behavior partly mediated relationships between sleep quality and BMI and that night eating patterns partly mediated the relationship between BMI and sleep quality. Trace and colleagues [28] highlighted that women from the Swedish Twin Study of Adults who reported binge eating behaviors (i.e., measured by two questions assessing history of ever eating large amounts of food accompanied by a subjective loss of control) were more likely to have poor sleep even after accounting for obesity status. These studies suggest a unique relationship between binge eating and sleep disturbance among non-clinical populations. Yeh and Brown [27] described their sample as being somewhat limited as the majority of their participants were well-educated, female, and on average healthy weight, likely due to college students making up a significant proportion of their sample. Furthermore, disordered eating assessment was limited to the Binge Eating Scale and did not specifically examine loss of control eating or quantifiable OBEs or SBEs, both of which are disordered eating patterns that also are prominent in community samples [29]. Similarly, the Trace and colleagues [28] study was limited in the generalizability of their female-only sample from Sweden and assessing disordered eating with two questions.

Overall, the literature suggests relationships between disordered eating behaviors and sleep disturbance; however, the studies tend to be limited to clinical, healthy-weight, female only, and/or college-aged samples and at times has relied on non-validated measures of sleep and disordered eating. The current study sought to add to the literature by utilizing validated measures for relevant constructs and by building upon Lawson and colleagues’ [23] method with a larger community sample and a broader measure of impact to functioning to help inform population-based assessment and treatment. Given previous research showing the relationship between sleep and depression [6, 7] and that Lawson and colleagues [23] found significant relationships between disordered eating, sleep, as well as mental- and physical-health functioning, the current study also included measures of depression and weight-related functioning. Weight-related functioning is a novel variable within the sleep and disordered eating literature that builds off the data showing relationships between sleep and weight [1] and quality of life [4]. Weight-related functioning measures individuals’ perceptions that their weight negatively impacts their functioning across several spheres of life [30]. Including the construct of weight-related functioning further extends previous results by drawing direct focus to how individuals perceive their weight may impact their lives. Based on the existing literature, it was hypothesized that:

1. A majority of individuals will report poor sleep quality.
2. Poorer sleep quality will be significantly and positively related to worse disordered eating, depression, and weight-related functioning.
3. Participants endorsing poor sleep quality will report significantly more problematic disordered eating, depression, and weight-related functioning compared to participants endorsing good sleep quality.
4. Following an analytical framework similar to Lawson and colleagues [23], it was hypothesized that patterns of poor sleep quality and greater disordered eating would be associated with greater depression and poorer weight-related functioning.

## Methods

### Participants

Adult participants (*N* = 648) were primarily female (*n* = 424, 65.4%), and the racial–ethnic distribution was: White non-Hispanic (72.7%), Asian (9.0%), Black (7.1%), White Hispanic (7.4%), Native Alaskan/American Indian (1.7%), Native Hawaiian/Pacific Islander (0.3%), Bi/multiracial (1.1%), and 0.8% of participants did not identify with any of the provided categories. The mean age and body mass index (BMI) were 37.5 (*SD* = 12.3, range 18–80) years and 27.3 (*SD* = 6.9, range 16.0–61.2) kg/m^2^, respectively. BMI was used as an independent variable, was calculated using self-reported height and weight (kg/m^2^), and the mean fell within the overweight range.

### Procedures

Participants utilized Amazon's Mechanical Turk (MTurk) online platform to complete an online survey on eating and health attitudes. Individuals were eligible if they were 18 years or older and spoke English. Participants were unable to skip questions but could choose to discontinue the survey at any time. Robust studies have indicated that the online platform generally produces reliable data [31–33]. Some data, however, also suggest the potential for threats to data quality [34–36]. Among those who completed at least one item beyond consenting, a number of steps were taken to ensure data quality. First, to be included, participants were required to respond correctly to all five different types of quality control questions. We also inspected data for illogical or impossible response patterns (e.g., required to provide height/weight twice, eliminated outliers beyond three times the standard deviation of the mean). Participants were paid 0.50 cents. This study received approval from the Human Investigation Committee (i.e., IRB), and all participants provided electronic informed consent.

### Measures

Eating Loss of Control Scale (ELOCS) [37] is a validated 18-item self-report scale assessing subjective experience of loss of control over eating in the past 28 days, with each item containing two parts. Part one assesses the frequency of specific LOC-eating events and part two assesses severity or the degree to which the person experienced feeling out of control (e.g., 0—*Not at all hard to stop* to 10—*Extremely hard to stop*). Severity scores are calculated by taking the average of the latter severity items. Higher scores are indicative of greater LOC-eating severity. Cronbach’s alpha for the current sample was 0.952.

Pittsburgh Sleep Quality Index (PSQI) [38] is a widely used and validated 19-item self-report measure of sleep quality during the past month that provides a global score (ranging from 0 to 21). Higher PSQI global scores indicate worse sleep quality, and global scores over 5 are categorized as meeting criteria for poor sleep quality. Cronbach’s alpha for the current sample was 0.782.

Sheehan Disability Scale (SDS) [30] is a validated self-report measure of functional impairment based on a specific topic. For this study, participants were asked to rate their level of impairment based on their “weight” in three spheres of life: 1. Work/School (paid/unpaid, with an option to opt out if not working due to an unrelated reason); 2. Social Life; and 3. Family Life/Home Responsibilities on a 0 (*Not at all*) to 10 (*Extremely*) scale. Scores range from 0 to 30 with higher scores indicating greater weight-related functional impairment. Cronbach’s alpha for the current sample was 0.910.

The Eating Disorder Examination Questionnaire—Version 17 (EDE-Q) [39] is a widely used and validated self-report measure of eating-disorder psychopathology and overeating behaviors during the past 28 days resulting in a Global Score. The Global Score can range from 0 to 6 with higher scores indicating greater severity of symptoms, and Cronbach’s alpha for the current sample was 0.877. Behaviors measured by this scale also include frequency of objective (OBEs) and subjective (SBEs) binge eating episodes.

Patient Health Questionnaire-2 (PHQ-2) [40] is a validated measure that assesses the core symptoms of a major depressive episode in two self-report questions. Responses are scored on a 0 (*Not at all*) to 3 (*Nearly every day*) scale. The PHQ-2 has strong construct and criterion validity with longer self-report measures of depression and independent structured interviews, respectively [41]. Higher scores suggest greater depression severity. Cronbach’s alpha for the current sample was 0.870.

Statistical Analyses. The current sample size exceeded recommended sample sizes based on a power-analysis using G*power. Data were screened and outliers beyond three standard deviations of the mean were removed. Pearson’s correlations were used to explore the relationship between sleep quality, BMI, demographic characteristics (age, sex, race/ethnicity), disordered eating symptoms, depression, and weight-related functioning. *T*-tests and analyses of covariance (ANCOVA) were used to test for mean group differences between participants categorized (based on the PSQI) as reporting good or poor sleep quality on measures of disordered eating symptoms, depression, and weight-related functioning. Multivariate regression modeling assessed the independent contributions of sleep quality, after adjusting for correlated variables, on depression (PHQ-2) and weight-related functioning (SDS). Data met assumptions for regression analyses, including normality, linearity, homoscedasticity, and absence of multicollinearity.

## Results

Of the overall participant group, the mean PSQI global score was 6.5 (*SD* = 4.0), which is above the established PSQI cutoff score (> 5) and indicative of poor sleep quality. Over half of the participants (52.9%, *n* = 343) endorsed poor sleep quality, and sleep duration for those meeting criteria for good sleep quality (*M* = 7.4 h per night, *SD* = 0.9) was significantly higher than those with poor sleep quality (*M* = 6.5 h per night, *SD* = 1.3), *t*(595.8) = 10.9, *p* < 0.001.

Table 1 describes the most commonly endorsed sleep problems as per the PSQI, occurring at least once weekly. Notably, 52.9% of the sample endorsed nighttime or early awakenings. The PSQI global score was correlated significantly with age (*r* = − 0.09, *p* = 0.02) and BMI (*r* = 0.20, *p* < 0.001). There were no significant differences between White and non-White participants, *t*(646) = 0.94, *p* = 0.35, whereas women (*M* = 6.8, *SD* = 4.1) reported significantly poorer sleep than men (*M* = 6.0, *SD* = 3.8), t(644) = − 2.67, *p* = 0.008.

**Table 1:** Self-reported sleep problems occurring at least once per week

Sleep Problem	Percentage endorsing ≥ 1/week
Nighttime or early awakening	52.9
Getting up to use bathroom	51.3
> 30 min to fall asleep	32.6
Too hot	26.4
Pain	18.1
Bad dreams	17.2
Too cold	17.1
Coughing/snoring	15.6
Excess weight	11.8
Other	8.7
Heartburn	8.6
Difficulty breathing	8.5
Needing/wanting to eat	7.6
Difficulty with CPAP machine	6.0
Need to be elevated	5.3

Table 2 reports the bivariate correlations of the PSQI global score with the other clinical variables as well as group differences between participants categorized as reporting good or poor sleep quality. PSQI global scores were significantly positively correlated with eating-disorder psychopathology, depression, and weight-related functioning. When considered as a group, participants with poor sleep quality endorsed higher levels of eating-disorder psychopathology, depression, and weight-related functioning. The largest effect sizes were observed for depression (large), followed by EDE-Q Global and ELOCS (medium–large), weight-related functioning (medium), and OBEs and SBEs (small–medium). Analyses of covariance (ANCOVA) were performed to adjust for age, BMI, and sex. After adjusting, the overall pattern of findings did not change; individuals with poor sleep endorsed significantly greater symptoms on all measures assessed.

**Table 2:** Means and standard deviations of clinical variables by group and bivariate correlations with PSQI

	Total Sample *N* = 648	Good Sleep Quality *n* = 305	Poor Sleep Quality *n* = 343	Independent samples *t* test	Effect size	ANCOVA (Age, BMI, Sex)	Effect size
	PSQI Global Score* (r)*	M (SD)	M (SD)	*T*	Cohen’s *d*	*F*	η^2^
EDE-Q—Global	0.39**	1.46 (1.21)	2.37 (1.35)	− 8.99***	0.71	48.42***	0.07
OBE Frequency	0.26**	1.02 (2.87)	2.63 (4.91)	− 5.16***	0.40	16.20***	0.03
SBE Frequency	0.19**	1.30 (3.85)	3.00 (5.42)	− 4.62***	0.36	13.41***	0.02
ELOCS	0.38**	1.55 (3.00)	1.81 (2.24)	− 9.12***	0.71	60.11***	0.09
PHQ-2	0.50**	0.67 (1.13)	2.00 (1.78)	− 11.46***	0.88	103.06***	0.14
SDS Total	0.33**	2.46 (4.32)	5.99 (6.68)	− 8.07***	0.62	41.85***	0.06

EDE-Q = Eating Disorder Examination Questionnaire; OBE = objective binge episode; SBE = subjective binge episode; ELOCS = Eating Loss of Control Scale; PHQ-2 = Patient Health Questionnaire 2 item; SDS = Sheehan Disability Scale

*p ≤ 0.05 **p ≤ 0.01 ***p ≤ 0.001

Table 3 summarizes the multivariate regression models predicting weight-related functioning (SDS) and depression (PHQ-2). Sleep quality independently predicted depression, as did disordered eating. Sleep quality did not independently predict weight-related functioning (trended, *p* = 0.056), although disordered eating did, with the exception of SBEs.

**Table 3:** Regression analyses predicting self-reported depression and weight-related functioning

	*B*	SE *B*	β	95% confidence interval		*p* value
				Lower bound	Upper bound	
Model 1: PHQ-2						
Age	− **0.01**	**0.01**	− **0.09**	− **0.02**	− **0.00**	**0.013**
BMI	0.00	0.01	0.00	− 0.02	0.02	0.913
Sex	− 0.08	0.12	− 0.02	− 0.30	0.15	0.511
EDE-Q-Global	**0.15**	**0.05**	**0.13**	**0.05**	**0.26**	**0.005**
SBE Frequency	**0.04**	**0.01**	**0.10**	**0.01**	**0.06**	**0.004**
OBE Frequency	− **0.04**	**0.02**	− **0.10**	**0.06**	**0.19**	**0.007**
ELOCS	**0.13**	**0.03**	**0.17**	**0.08**	**0.21**	**0.000**
PSQI Global	**0.16**	**0.02**	**0.38**	**0.13**	**0.19**	**0.000**
Model 2: SDS						
Age	− **0.03**	**0.02**	− **0.06**	− **0.06**	**0.00**	**0.049**
BMI	**0.10**	**0.03**	**0.13**	**0.05**	**0.17**	**0.000**
Sex	− **10.17**	**0.40**	− **0.09**	− **10.94**	− **0.40**	**0.003**
EDE-Q-Global	**0.99**	**0.18**	**0.23**	**0.63**	**10.35**	**0.000**
SBE Frequency	0.06	0.04	0.05	− 0.02	0.15	0.121
OBE Frequency	**0.13**	**0.05**	**0.09**	**0.03**	**0.24**	**0.010**
ELOCS	**0.85**	**0.11**	**0.31**	**0.63**	**10.08**	**0.000**
PSQI Global	0.10	0.05	0.07	− 0.00	0.20	0.056

Bold values indicate a significant* p*-value

PHQ-2 = Patient Health Questionnaire 2 item; BMI = Body Mass Index; EDE-Q = Eating Disorder Examination Questionnaire; SBE = Subjective binge episode; OBE = Objective binge episode; ELOCS = Eating Loss of Control Scale; PSQI = Pittsburgh Sleep Quality Index; SDS = Sheehan Disability Scale

## Discussion

Most literature investigating connections between sleep quality and disordered eating focuses on clinical populations. To the best of our knowledge, this is the first study to assess relationships between sleep behaviors and disordered eating that also accounts for impact on weight-related functioning in a large community sample. Overall, poor sleep quality was common and significantly associated with disordered eating, depression, and impaired weight-related functioning. The strength of these relationships was maintained even after accounting for participants’ age, BMI, and sex.

Regarding disordered sleep, over half the *community* sample (52.9%) endorsed sleep quality above the *clinical* cutoff for poor sleep quality, and participants reported an average sleep duration of 6.5 h nightly. This finding is comparable to other studies of disordered sleep in the general population (51%) [27] and surprisingly close to estimates of sleep problem prevalence in those with diagnosed eating disorders (57%) [22] and a clinical population following sleeve gastrectomy (58.6%) [23]. The rate similarities also provide support for the validity for the current data collected via MTurk. In both the Lawson and colleagues’ paper [23] and the current, mean sleep durations fell below the National Sleep Foundation recommendation of 7–9 h per night for adults [41]. Prior and current results suggest that primary barriers to sleep include difficulty falling asleep and night time awakenings [22, 23]. Pain and waking due to excess weight, however, have been found to be more prevalent sleep-related concerns in clinical populations [23]. Current findings indicate areas of sleep intervention may differ somewhat depending on the population, encouraging a precision medicine approach. For example, improving sleep onset within community samples versus pain management within clinical samples. The prevalence of difficulty falling asleep across diverse samples leads to speculation of environmental issues that may exacerbate that concern, such as technology use [42], shift work [43], and the societal pressure to work around the clock [44].

In addition to the relatively high incidence of poor sleep quality, noteworthy findings include the relationship between sleep quality and disordered eating within a purely community sample. The current results align with a recent meta-analysis that binge eating was specifically related to worse sleep [24] but also expand to other related symptoms that may be more widespread, including overall eating psychopathology, loss of control eating, and subjective binge episodes. Yeh & Brown [27] found people with overweight and obesity reported delayed sleep onset and reduced sleep duration. Researchers speculated that the chronic delayed sleep onset in individuals with excess weight may provide more time for disordered eating and perhaps much of this behavior occurs in solitude [14]. In a population-based study of female twins, binge-eating was related to insomnia and was a risk factor for psychiatric and medical disorders [45]. In a study of both ADHD and obesity, binge eating episodes, consuming large amounts of food, waking up at night, and eating in secret were more prevalent in individuals with obesity and ADHD than in comparison with those with obesity without ADHD, though both groups had evidence of OBEs [46]. Such findings allude to the presence of complex patterns of comorbidities and disordered patterns of both sleep and eating behaviors in clinical populations; however, the current study findings suggest similar patterns also among community individuals that may be overlooked as they are not seeking treatment for disordered eating.

Sleep quality also was associated with weight-related functioning and depression, even after controlling for age, BMI, and sex. Participants with poor sleep quality endorsed significantly worse weight-related functioning, regardless of BMI, whereas disordered eating significantly predicted weight-related functioning in regression analyses and sleep did not (i.e., trended towards significance). Future research is needed to consider the possible mediation or moderation relationships among sleep, disordered eating, and weight-related functioning. Though this is the only known study to look at the relationships between these variables simultaneously in this specific way, past research has considered portions of these relationships, mostly in clinical populations. Studies from the National Health and Wellness Survey (NHWS) of individuals meeting criteria for various eating disorders also considered functional impairment relative to healthy comparison groups. Individuals with BED exhibited significant impairment across all domains from health-related quality of life (HRQOL) to work-related impairment [47]. If our results generalize in larger population studies, there are important research and practical implications for clinicians, epidemiologists, policymakers, and other stakeholders. Poor sleep quality, as well as obesity and disordered eating can interfere with both overall quality of life and contribute to functional impairment across multiple life domains (e.g., work, social). There also is an additive effect, where individuals with physical or mental health comorbidities have even greater deficits in functioning when their sleep is poor [48].

### Clinical implications

This research provides a cross-sectional evaluation regarding the complicated relationships between disordered eating, sleep, and depression and weight-related functioning in a community population. This is an important step in clarifying the multifaceted relationship among these vital and modifiable health behaviors and results may inform next steps in research, including longitudinal, randomized controlled studies to evaluate treatment for these concerns. These results also offer important information for clinicians across mental health and medical disciplines as they assess community populations and subsequently treat multiple concerns regarding disordered eating, weight, sleep and mental health. Furthermore, interventions designed to treat one of these concerns also may benefit from considering the comorbidity of concerns and their impact on individual’s functioning in across life domains.

### Strengths and limits

The novel combination of sleep, eating, and weight-related functioning is a strength of this study. Furthermore, the sample size is large, community-based, and participants were assessed with standardized measures [24]. Participants also represented a broad range of ages, BMIs, and men and women were included. Findings of the current study do have limitations. Participants were primarily White non Hispanic. All the measures were self-reported from a convenience sample collected via Mturk. Multiple and rigorous assessments of data validity were followed; however, replication within other community samples will be required. No objective measures of health behavioral data were collected. Some participants reported problems with sleep, such as difficulty breathing (8.5%) and difficulty with CPAP machine (6%). Given that sleep concerns such as obstructive sleep apnea are associated with obesity [49], it could serve as a control in future research. Since the data were cross-sectional, causal or directional relationships cannot be interpreted from this study, it also is feasible that bi-directional relationships exists between disordered eating and sleep such that a feedback loop is created further complicating conclusions about directionality [50]. Moreover, assessing patterns or changes in sleep quality and eating behaviors over time or in the context of various environmental stressors was not possible given the study design. These data were collected prior to COVID-19; therefore, these findings may underestimate the current incidence of depressive symptoms, eating, and sleep concerns as recent research has found increased sleep problems [51]. Our research captured weight-related functioning across multiple domains in an adult population, while future research could focus on age groups known to be vulnerable to disordered sleep and eating, such as in the important development period of adolescence [22].

Evaluating the relationship between sleep behaviors, disordered eating, and weight-related functioning among a large diverse participant group may help inform population-based assessment and treatment. In general, there is a call for more research to better understand and address the needs of diverse community samples that may share similarities to clinical samples regarding their sleep quantity, sleep quality, and maladaptive eating [47, 48]. Future research requires objective data as well as clinical assessment to gather a complete picture of depressive symptoms, sleep impairments, and disordered eating with a more racially diverse sample. In future studies, more detail about specific sleep concerns or related medical diagnoses, such as OSA, could also be gathered due to the high prevalence for individuals with obesity [49]. From there, understanding the implications for weight-related functioning could be more fully understood, informing both assessment and evidence-based treatment approaches.

### What is already known about the subject

- Sleep quality is linked to obesity, depression, quality of life, and disordered eating.
- Most research is limited to clinical populations.
- It is important to investigate and understand these variables within more generalizable community samples to assess the presence of modifiable health behaviors.

### What this study adds

- This research suggests the relevance of significant sleep concerns in the general population as well as shedding light on differing sleep concerns across clinical and community samples.
- The results showed relationships between poorer sleep and worse disordered eating behaviors and weight-related functioning, regardless of BMI.
- The current paper sets the stage for future research to delve more deeply into the complicated relationships between disordered eating, sleep, and mental health and weight-related functioning as well as assessing and treating these concerns in community populations.

## Data Availability

The data generated during and/or analyzed during the current study are available on reasonable request.
